# Increasing women’s engagement in vector control: a report from Accelerate To Equal project workshops

**DOI:** 10.1186/s12936-018-2477-0

**Published:** 2018-09-10

**Authors:** Kacey C. Ernst, Erika Barrett, Accelerate to Equal Kenyan and Indonesian Working Groups, Hawa Abdillahi, Vitalis Akora, Helen Amegbletor, Rosemary Ayiera, Guyah Bernard, Zeddy Bore, Chuchu Cowan, Abraham Rono, James Sang, Tom Wabwire, Caroline Murerwa, Kiambo Njagi, Peter Njoroge Ng’ang’a, Lilian Odipo Omondi, Caroline Otieno, Bruno Otsyula, Eggi Arguni, Pak Triwibowo Ambargarjito, Mike Bangs, Claus Bogh, Elisabeth Dodok, Martha Hebi, Elsa Herdiana, Sarah Hobgen, Say Piau Lim, Baning Rahayujati, Tri Ramadhani, Susi Soviana, Asep Suryaman, Susilowati Tana, Marieti Wala, Elizabeth Hoswell, Mary H. Hayden

**Affiliations:** 1 Mel and Enid Zuckerman College of Public Health, University of Arizona, Tucson, AZ USA; 2 National Center for Atmospheric Research, Boulder, CO USA; 3 Trauma, Health and Hazards Center, University of Colorado, Colorado Springs, CO USA

**Keywords:** Gender equity, Vector control, Women, Stakeholder workshop

## Abstract

Workshops with academic, national and local government, and community stakeholders were held in Kenya (2017) and Indonesia (2018) to understand the role and perceptions of women in vector control and to identify strategies for accelerating involvement of women in sustained support for vector control interventions at multiple levels/sectors.

## Background

Involving women in programmes to improve the well-being of their family and community can improve gender equity and lead to more sustainable programming [1–3]. Concerted efforts have been made in sectors such as forestry and agriculture to increase women’s participation and leadership roles. Evidence suggests that when policies and programming explicitly empower women in these sectors, overall improvements in food security and nutrition and sustainable resource management have been achieved [4]. Yet gender mainstreaming, the process by which a gender-based lens is focused on the preparation, design, implementation, and evaluation of programs and policies, has not been systematically incorporated into vector control. Prior to broad-scale implementation of gender-based policies, it is critical to develop an understanding of the barriers and opportunities to engaging women more fully in vector control programmes. This must be done across all potential levels of engagement including: the household-level in which women are empowered to carry out vector-control activities within their own homes, the community-level in which women carry out paid or unpaid vector control activities with residents to build collective action at the local level, and finally, the professional level in which women engage in regional or national-level vector-control efforts.

Following implementation of survey and field-based research in Indonesia and Kenya, collaborators from the “Accelerate to Equal: Engaging Women in Vector Control” project held stakeholder workshops in Lake Naivasha, Kenya in December 2017 and Bali, Indonesia in March 2018 to understand the current role and perceptions of women in vector control, and to identify potential strategies for accelerating involvement of women in sustained support for malaria control interventions at multiple levels and sectors. In previous work, current vector control professionals identified lack of awareness of opportunities, cultural barriers, and household obligations as leading barriers to engagement in vector control efforts in the community [5]. Addressing cultural barriers and reducing household obligations were perceived as being the most difficult to address.

The stakeholder workshops engaged multi-sectoral stakeholders to align findings from field and survey investigations in Kenya and Indonesia with current vector control initiatives. Stakeholder input was sought to refine models for framing the barriers and opportunities for women in vector control. They were also used to identify specific steps that could be implemented to increase women’s participation in vector-control activities at household, community, and professional levels. Decision-makers and other stakeholders came together from the national level (ministries of health and environment, as well as women’s empowerment groups and similar stakeholders in Kenya) and from the local level (vector control, women’s empowerment, women community leaders) to a provide a mechanism for capacity building, which included a researcher-stakeholder dialogue and promotion of coordination among local and national practitioners/decision-makers.

## Methods

### Participant recruitment

Eligible participants fell within one or both of two categories; (1) an individual currently working in a vector-control programme and/or (2) an individual currently working in gender equity or women’s empowerment. Two stage-recruitment was conducted. First, direct recruitment was conducted via an email invitation to contacts within the health ministries, non-governmental organizations, and commercial organizations. Upon confirmation of participation, individuals working in community programmes were asked to invite at least one community partner to participate in the workshop to provide both a top-down and bottom-up perspective of engaging women in the community.

### Workshop implementation

A semi-structured approach was taken for the workshops. Both workshops began with introductions of all participants and organizers. To provide all participants with common knowledge, research results of previously conducted household surveys, focus groups, and key informant interviews were briefly presented. The meeting then transitioned into working groups that discussed benefits, barriers, and strategies to engage women in vector control. Specific implementations are provided below for each country.

#### Kenya

The “Accelerate to Equal: Engaging Women in Vector Control workshop” was held in Lake Naivasha, Kenya on December 4, 2017.

After the initial introductions and research presentation, participants broke into groups of approximately 4–5 participants to discuss and identify key benefits and barriers to women working in vector control at three levels; household (H), community (C), and professional (A). After a report back from each group, participants were then randomly assigned into new groups to brainstorm solutions and strategies to overcome the identified barriers. The solutions were displayed for the whole group, and the group then selected the top strategy focus for each level (H, C, A) with one short-term and one long-term strategy. Participants subsequently self-selected into working groups to identify key activities, outputs, and resources needed for each strategy. The small groups then presented their ideas to the broader group for feedback and discussed challenges to implementation of the proposed solutions.

#### Indonesia

The “Accelerate to Equal: Engaging Women in Vector Control workshop” was held in Bali, Indonesia on March 5th and 6th, 2018.

After the initial introductions and research presentation, the organizers used a modified technology of participation (ToP) (http://top-facilitation.com/empowering-tools/consensus-workshop-method/) workshop strategy to build consensus about the benefits and barriers to women working in vector control for each level (H, C, A). After consensus was achieved among the large group, participants then went back to their small groups to identify solutions to the barriers at each level. After a report back, the participants then self-selected into working groups for interventions and strategies at each level (H, C, A). All groups developed components that contributed to a larger system. While this was not the initial intention of the workshop, there were enough similar themes and discussion threads that a single system could be created that would facilitate a model for community-led vector control with a female-centric focus. Professional translators provided simultaneous translation to facilitate exchange among participants who spoke only Bahasa or English. The small groups then presented their ideas to the broader group for feedback and discussed challenges to implementation of the proposed solutions.

### Identification of themes

An iterative process was conducted between participants and workshop facilitators, to ensure integrity of the report. All notes were collated for Kenya and Indonesia. Notes from Kenya were all recorded in English. Some notes from Indonesia were written in Bahasa and coded on site with the professional translators assisting during the workshop. Notes were provided back to workshop participants for a 2-week comment period. After notes were finalized by all participating individuals, three individuals coded the resulting English language workshop notes; KE, MH, and EB. Each workshop was coded separately for themes in benefits, barriers, and strategies to engage women in vector control. Resulting themes were sent to participating workgroups for clarification. Themes from the two groups were then collated and coded into overarching themes.

### Workshop outcomes

#### Overview

**Fig. 1:**
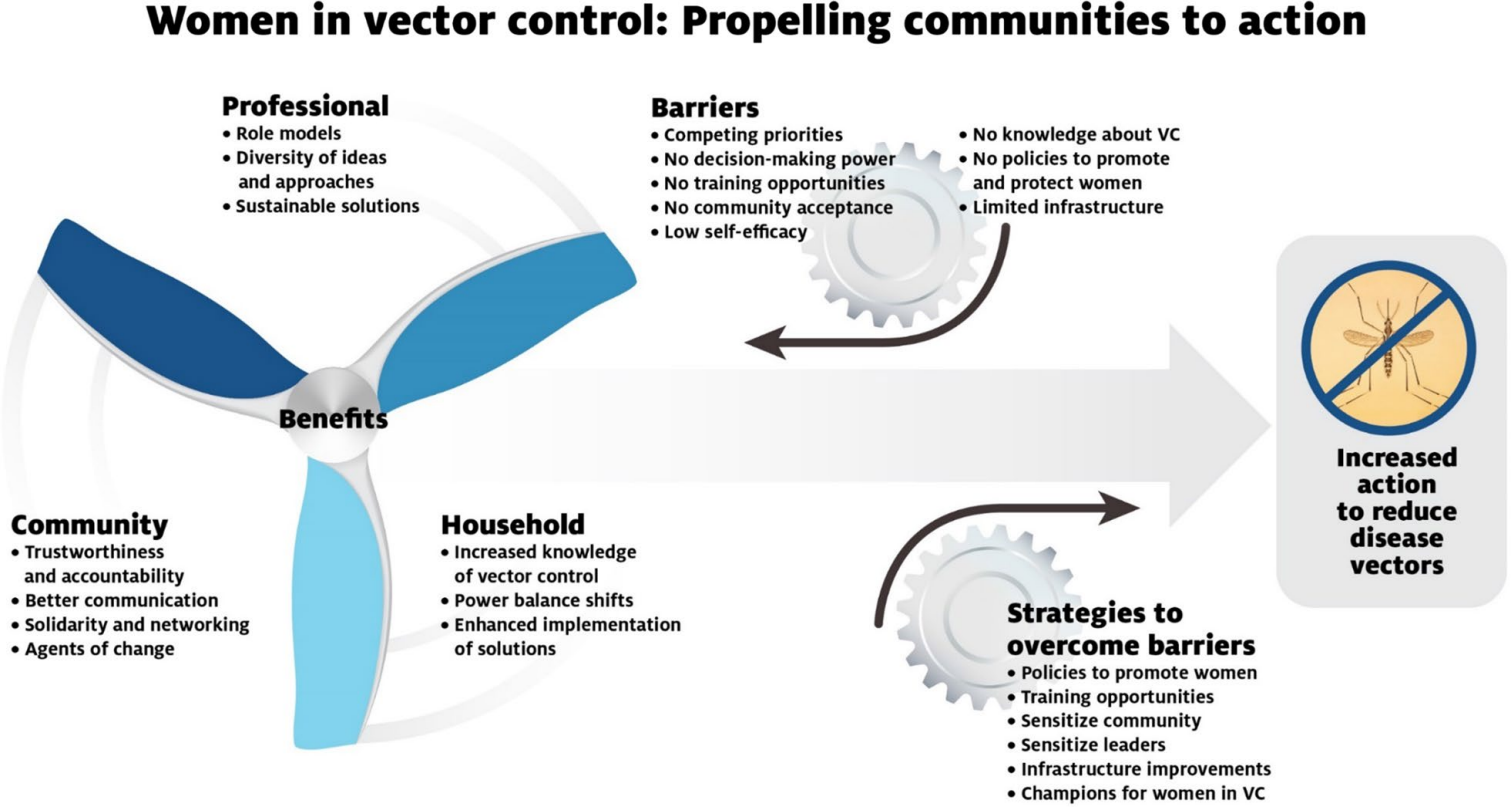
Summary of benefits, barriers, and strategies generated by workshop participants to engage women as agents of change in vector control. The benefits of women in vector control will drive action, but barriers must be overcome with specific gender-focused strategies to achieve success

A framework for the benefits, barriers, and strategies to engage women in vector control activities was developed (Fig. 1). Benefits were identified for engaging women at the household, community and professional levels. Barriers and strategies were identified that crossed all levels.

#### Benefits

Both working groups concluded that engaging women in vector control would deliver two broad categories of benefits; first, it was widely held that engaging women at all levels would lead to more sustainable vector-control strategies; and second, there were potential benefits for the women themselves through economic and/or social empowerment. Women were perceived as having more knowledge about their household, including both the household members and the household environment. As participants from Kenya stated, “when it comes to decisions on vector control, women make faster decisions because of a better understanding of household and surroundings” (Table 1). They were also perceived as being the ones who would most likely implement household level measures of protection. At the community level, women were perceived as having better communication skills, and being more accountable and trustworthy than male counterparts. They were more likely to take part in group activities and have networks on which to rely for implementation projects. At the professional level, including more women was perceived as being important to generate a diversity of ideas. Stakeholders also reported the importance of having women in leadership roles to mentor and inspire the next generation (Fig. 1, Table 1).

**Table 1:** Key benefit themes of engaging women in vector control at household, community, and professional levels

Household	Community	Professional
Enhancing women’s knowledge will increase household awareness of vectors and the diseases they carry As the household caretakers, women will better integrate appropriate vector control into household activities Women have a greater vested awareness of and interest in family well-being Engagement in paid vector control activities shifts the household power balance towards equality	Women are more trustworthy and accountable which enhances community buy-in Women are better communicators which increases visibility and acceptance of proposed programs Improved solidarity and networking among women strengthens community organizations Women act as agents of change to prioritize and improve overall community health	More comprehensive representation in programmes and policies leads to diversity of ideas and approaches Women understand the community and are more likely to administer programmes that are accepted and sustained When breaking the glass ceiling, women serve as role models for enhanced gender equity

#### Barriers

Despite the significant benefits, stakeholders also reinforced the idea that there were significant barriers to women’s participation. It was recognized that women’s roles in vector control would be impeded by cultural norms and competing priorities as primary caretakers of the household (Fig. 1, Table 2). Lack of voice in the community was also considered a significant barrier. As also stated in Kenya, “they [women] are not involved in decision-making processes. The chief calls a meeting, and the men come. The women miss these meetings”. Additional barriers included gaps in policy, specifically addressing women’s needs as vector-control personnel, resource constraints on carrying out action, lack of awareness and knowledge about effective strategies to control vectors, and no defined career paths or mentors for women in vector control (Fig. 1, Table 2).

**Table 2:** Key barriers to engaging women in vector control at household, community, and professional levels

Household	Community	Professional
Women lack the autonomy to make decisions about allocation of household resources Self-efficacy in women is low and may impede action. Women have decreased ability to act on economic improvement Competing household priorities may reduce time available for vector control activities Lower literacy and numeracy skills may reduce access to information hindering uptake and implementation of vector control	Lack of community acceptance of women’s involvement in vector control Mobility in community may be limited due to religious and cultural beliefs Top down patriarchal power structure reduces women’s ability to act as decision-makers Lack of prioritization to use limited resources on vector control Community lacks self-efficacy to control vector borne diseases Lack of available training opportunities for women to acquire necessary vector control skills	Lack of policies to promote and protect women in careers in vector control Inability to access career opportunities due to lower education, few female role models and lack of awareness of career opportunities Limited infrastructure designed to accommodate specific needs of women

#### Strategies

**Fig. 2:**
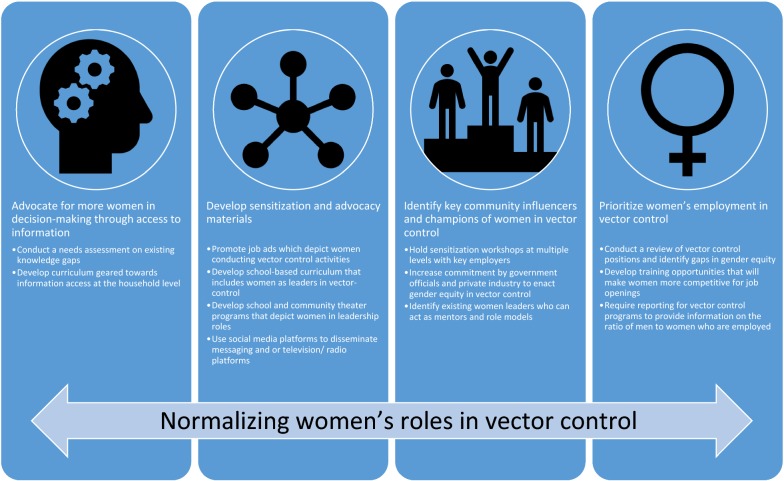
Strategies to overcome barriers to women engaging in vector control activities

Strategies were recommended within four domains. First, to advocate for more women in decision-making through access to information. Women are more likely to play a key role in the decision-making process when they are informed about vector control strategies. Determining key gaps in knowledge and understanding and providing curriculum to fill those gaps at a community level is one proposed strategy. This should be coupled with sensitization materials that depict women engaging in vector control to overcome cultural norms that may prevent women from engaging. Role models and champions should be identified that can reinforce the importance of women as leaders in vector control. Finally, existing programmes should review their current policies for recruitment to ensure they are targeting women. Several specific strategies that the groups considered had high potential for success included conducting systematic assessments of gender gaps in current programmes and developing metrics to track progress, enhancing women’s knowledge about vector control through community-delivered curriculum, using multimedia strategies to normalize women’s role in vector control, and developing mentoring and training programmes for women (Fig. 2).

Participants from both workshops suggested creating a repository of standard tools and templates that were flexible enough to be tailored to specific community needs. Participants felt that no one-size would fit all for solutions and that different disease systems were unique and should be addressed as such; i.e., engaging women in different arenas of vector control will differ by disease model and vector of interest.

Workshop attendees felt that engagement of women should address the secondary benefit of empowering women by providing some incentives, which could focus on economic benefits for participation, recognition at the community level, or even tokens of appreciation.

Participants noted that specific interventions were deemed secondary to providing a system that could be developed to flexibly adapt to new interventions as they became available so that a monumental effort to try something new is not necessary with each intervention. Women were perceived as critical to implementing new strategies by building community consensus and awareness.

**Fig. 3:**
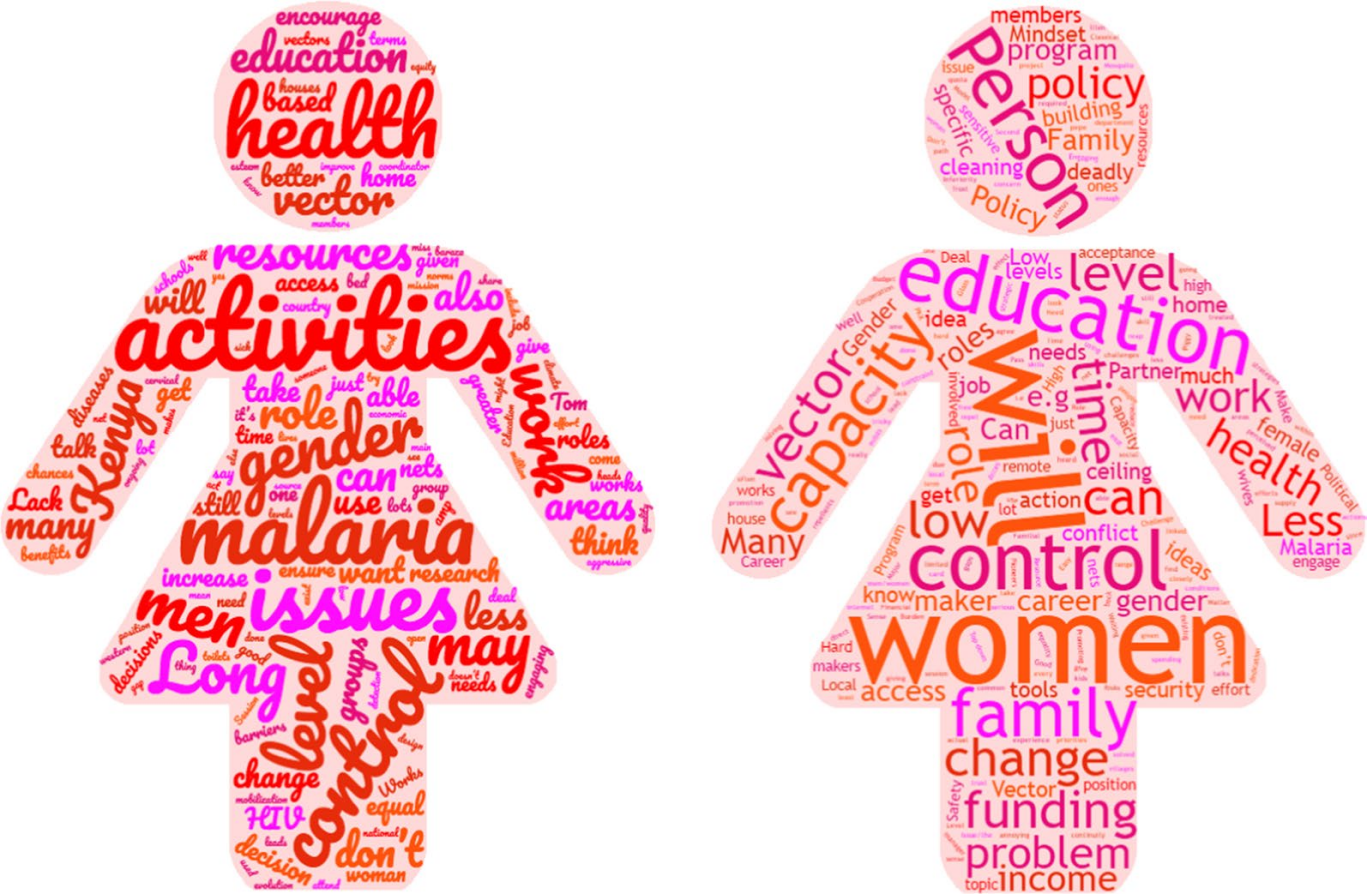
Word clouds generated from Kenya and Indonesia workshops

As a culminating activity, both Kenyan and Indonesian participants generated word clouds of key words that represented important workshop concepts (Fig. 3).

## Conclusion

Women play integral roles in their communities as educators, caregivers, and agents of change. These roles highlight the importance of engaging women in effectively designing and implementing culturally acceptable and sustainable vector control interventions and empowering them as key decision-makers from the outset. Women are instrumental in building community awareness and acceptance of interventions and will do so more efficiently when they combine science-based knowledge of the disease systems being addressed with their local knowledge of the community toward prioritizing and improving health overall in their communities. Empowering women at household, community and programmatic levels to fully participate in vector control will promote gender equity towards a goal of policy-driven and sustainable improvements in all sectors.

## Abbreviations

H: household
C: community
A: professional
ToP: technology of participation

## References

[1] Elmendorf ML, Isely RB. Public and private roles of women in water supply and sanitation programs. Society for Applied Anthropology. 1983. https://www.jstor.org/stable/44126002.

[2] Rahman SA (2008). Women’s involvement in agriculture in northern and southern Kaduna State, Nigeria. J Gend Stud.

[3] Agarwal B (2009). Gender and forest conservation: the impact of women’s participation in community forest governance. Ecol Econ.

[4] Food and Agriculture Organization of the United Nations. Women in forestry: challenges and opportunities. http://www.fao.org/3/a-i3924e.pdf. Accessed Aug 2018.

[5] Hayden MH, Barrett E, Bernard G, Toko EN, Agawo M, Okello AM (2018). Barriers and opportunities to advancing women in leadership roles in vector control: perspectives from a stakeholder survey. Am J Trop Med Hyg.

